# Protective effects of combination of *Stauntonia hexaphylla* and *Cornus officinalis* on testosterone-induced benign prostatic hyperplasia through inhibition of 5α- reductase type 2 and induced cell apoptosis

**DOI:** 10.1371/journal.pone.0236879

**Published:** 2020-08-13

**Authors:** Shanika Karunasagara, Geum-Lan Hong, Da-Young Jung, Kyung-Hyun Kim, Kyoungwon Cho, Ju-Young Jung

**Affiliations:** 1 Department of Veterinary Medicine & Institute of Veterinary Science, Chungnam National University, Daejeon, Republic of Korea; 2 Department of Pharmacy, College of Pharmacy, Chungnam National University, Daejeon, Republic of Korea; University of Hyderabad, INDIA

## Abstract

Benign prostatic hyperplasia (BPH) is a progressive pathological condition associated with proliferation of prostatic tissues, prostate enlargement, and lower-urinary tract symptoms. However, the mechanism underlying the pathogenesis of BPH is unclear. The aim of this study was to investigate the protective effects of a combination of *Stauntonia hexaphylla* and *Cornus officinalis* (SC extract) on a testosterone propionate (TP)-induced BPH model. The effect of SC extract was examined in a TP-induced human prostate adenocarcinoma cell line. Male Sprague-Dawley rats were randomly divided into 5 groups (n = 6) for *in vivo* experiments. To induce BPH, all rats, except those in the control group, were administered daily with subcutaneous injections of TP (5 mg/kg) and orally treated with appropriate phosphate buffered saline/drugs (finasteride/saw palmetto/SC extract) for 4 consecutive weeks. SC extract significantly downregulated the androgen receptor (AR), prostate specific antigen (PSA), and 5α-reductase type 2 in TP-induced BPH *in vitro*. In *in vivo* experiments, SC extract significantly reduced prostate weight, size, serum testosterone, and dihydrotestosterone (DHT) levels. Histologically, SC extract markedly recovered TP-induced abnormalities and reduced prostatic hyperplasia, thereby improving the histo-architecture of TP-induced BPH rats. SC extract also significantly downregulated AR and PSA expression, as assayed using immunoblotting. Immunostaining revealed that SC extract markedly reduced the 5α-reductase type 2 and significantly downregulated the expression of proliferating cell nuclear antigen. In addition, immunoblotting of B-cell lymphoma 2 (Bcl-2) family proteins indicated that SC extract significantly downregulated anti-apoptotic Bcl-2 and markedly upregulated pro-apoptotic B cell lymphoma-associated X (Bax) expression. Furthermore, SC treatment significantly decreased the Bcl-2/Bax ratio, indicating induced prostate cell apoptosis in TP-induced BPH rats. Thus, our findings demonstrated that SC extract protects against BPH by inhibiting 5α-reductase type 2 and inducing prostate cell apoptosis. Therefore, SC extract might be useful in the clinical treatment of BPH.

## Introduction

Natural products from herbal plants are frequently used in traditional medicine, and are organized as an alternative treatment option for different kinds of medical problems. *S*. *hexaphylla (Thunb*.*) Decne*. (*S*. hexaphylla) belongs to the family Lardizabalaceae, native to Southern Japan, Korea, and China, and has been used in medicine owing to its analgesic, sedative, diuretic, and anti-cancer properties [1]. Previous studies have reported that *S*. *hexaphylla* is a precious herbal medicine due to its anti-diabetic, anti-inflammatory, and antioxidant effects [2–6]. *Cornus officinalis* Sieb et Zucc (*C*. *officinalis*) of the family Cornaceae is native to Korea, Japan, and China, and owing to its chemical constituents, displays diverse pharmacological activities, such as hypoglycemic activity, antioxidant, anti-inflammatory, and anticancer activity [7, 8]. In this study, we investigated the protective effect of a 9:1 mixture of *S*. *hexaphylla* and *C*. *officinalis*, called the SC extract. According to their pharmacological properties and a prior report [9] together with our previous study [10], a combination of these two plants may benefit from a synergistic effect and consequently promises to be an effective treatment for BPH.

Benign prostatic hyperplasia (BPH) is the most frequent, non-cutaneous form of cancer among elderly men and is characterized by progressive glandular and stromal tissue hyperplasia, which leads to an enlarged prostate [11]. The rapid growth of stromal and epithelial elements results in BPH in the prostate, along with lower urinary tract symptoms (LUTS), including obstructive symptoms such as hesitancy, poor intermittent stream, feeling of incomplete bladder emptying, and irritative symptoms such as increased frequency, urgency, and nocturia [12, 13]. A previous report proved that approximately one-third of men older than 40 years showed symptoms related to LUTS, and its prevalence increases with age [14]. Androgens, especially testosterone-related hormones, are the major contributing factors in the development and progression of BPH [15]. Dihydrotestosterone (DHT) is an active metabolic product of the conversion of testosterone by 5α-reductase [16]. The growth of the prostate gland depends on DHT, which, together with aging, results in hyperplasia of prostatic cells [17].

5α-reductase type 2 inhibitors (5ARIs) and α-adrenergic blockers are typical therapeutic agents for the treatment of BPH. 5ARIs restrain the action of 5α-reductase type 2, thereby reducing DHT production and reducing prostate size [18]. In addition, α-adrenergic blockers relax the smooth muscles in the prostate and neck area of the bladder, making urination easier [19]. Finasteride, a drug that acts as a 5ARI, has been frequently used as a treatment for BPH [20]. Furthermore, saw palmetto has been widely used as a phytotherapeutic remedy for urinary dysfunction due to its 5ARI effect [21, 22]. Saw palmetto has also been reported to have antioxidant activity, along with inhibitory activity of the production of reactive oxygen species (ROS) under androgen stimulation [23, 24]. In this study, saw palmetto was used as a phytotherapeutic positive control. However, these drugs have been known to induce adverse effects during long-term administration. Therefore, many researchers have tried to investigate alternative therapeutic agents for BPH using phytotherapeutics, which are safer and less toxic than chemical agents [25, 26].

Cell proliferation and apoptosis are naturally occupying cellular activities in normal cell growth; however, in BPH states, the adult prostate continues to grow. Apoptosis is the molecular mechanism primarily responsible for the programed elimination of cells, which accompanies the regulation of organ size. Cell growth in the normal prostate is regulated by the balance between apoptotic and proliferative activity. Disruption of the molecular mechanisms that regulate these processes may lead to abnormal growth of the gland, resulting in BPH [27]. Hence, induction of apoptosis and inhibition of cell proliferation are probable treatments for BPH [28]. Therefore, this study aimed to investigate the protective effect of the SC extract in testosterone-induced BPH *in vitro* and *in vivo*. Furthermore, we examined the role of SC extract in apoptosis induction under BPH conditions.

## Materials and methods

### Plant material

The SC extract consisted of *S*. *hexaphylla* leaves (Lot number: 20181116) and *C*. *officinalis* Siebold & Zucc fruits (Lot number: 20181112) mixed in a 9:1 ratio. The leaves of *S*. *hexaphylla* were harvested in the area of Goheung-gun, Jeollanam-do, Korea, and dried at 60°C for 12 h. Next, dried leaves were extracted with 70% ethanol at 75°C for 12 h and then spray-dried with 30% dextrin. The dried fruit of *C*. *officinalis* Siebold & Zucc was harvested in the area of Gurye-gun, Jeollanam-do, Korea. Dried fruit was extracted with 70% ethanol at 75°C for 12 h, and then spray dried with 50% dextrin. The powder form of this SC extract (CKDHC-P29) was provided by Chong Kun Dang Healthcare (Seoul, Korea) for this study and dissolved in dimethyl sulfoxide (DMSO, Sigma-Aldrich, UK) and phosphate buffered saline (PBS) to prepare the appropriate SC extracts for *in vitro* and *in vivo* experiments, respectively.

### Drugs and chemicals

The reference compounds, hederacoside D (purity ≥ 98.0%, ChemFace, Wuhan, China) and morroniside (purity ≥98.0%, ChemFace, Wuhan, China) were analyzed in the SC extract. Reagents including acetonitrile, methanol, and ethanol were purchased from Burdick & Jackson (Muskegon, MI, USA) and formic acid (HPLC grade) was purchased from Sigma-Aldrich (St. Louis, Mo, USA). Water was purified using a Milli-Q system (Sinhan, Seoul, Korea). Saw palmetto was obtained from Mitsuya Boeki Ltd (Osaka, Japan) and dissolved in DMSO and PBS for *in vitro* and *in vivo* experiments, respectively. Testosterone propionate (TP, Tokyo Chemical Industry Co., Ltd, Tokyo, Japan) was dissolved in ethanol and corn oil for the *in vitro* and *in vivo* studies, respectively.

### High-performance liquid chromatography (HPLC) of SC extract

HPLC was used to identify the compounds, hederacoside D (C_53_H_86_O_22)_ and morroniside (C_17_H_25_O_11_), in the SC extract. The composition of the SC extract was analyzed using HPLC equipped with a photodiode array detector (PDA, Berlin, Germany) using an Agilent Zorbax eclipse plus C18 column (4.6 × 250 mm, 5 μm). Elution was performed using a linear gradient from 12 to 0.1% formic acid in acetonitrile for detection of hederacoside D and morroniside, and the injection volume was 10 μL. The PDA detector was set at 205 nm and 240 nm for the appropriate detection of hederacoside D (205 nm) and morroniside (240 nm).

### Cell viability assay

The androgen-sensitive human prostate adenocarcinoma cells (LNCaP) were purchased from the American Type Culture Collection (ATCC, Manassas, VA, USA) and cultured in Roswell Park Memorial Institute (RPMI)-1640 medium (Gibco, NY, USA) supplemented with 10% FBS (Gibco), 100 mg/mL penicillin (Gibco), and 100 mg/mL streptomycin (Gibco). The cells were maintained in a 5% CO_2_ incubator at 37 ^o^C with the replacement of the medium every 48 h. Cytotoxicity of the SC extract on LNCaP cells was evaluated by using an MTT assay, using EZ-Cytox Cell Viability assay kit (Biomax, Seoul, Korea), according to the manufacturer’s instructions. Briefly, cells were seeded at 1×10^4^ cells per well in a 96-well plate and incubated at 37°C in a 5% CO_2_ atmosphere for 24 h. Cells were then treated with concentrations of SC extract (0, 5, 10, 25, 50, 100 μg/mL) for 72 h. Absorbance was evaluated using a microplate reader (BIO-TEK, Senergy HT), and cell viability was calculated as: 100% × (OD_450nm_ of SC group/ OD_450nm_ of control group).

### Animal experimental design

The experimental protocols were approved (CNU-01108) by the International Animal Ethics Committee at Chungnam National University. Six-week-old male Sprague-Dawley (SD) rats were purchased from Orient Bio (Gyeonggi-do, Korea) and acclimated in a specific-pathogen-free (SPF) animal facility under controlled temperature, humidity, and photoperiod (22 ± 2 ^o^C, 55 ± 5%, and 12 h light/dark cycle, respectively) for 1 week before the experiment. All animals were fed standard chow and water *ad libitum*. During the experimental period, rats were visually inspected for their health and well-being daily and the in-house standards were maintained, including the removal of bedding with full cage changes twice a week. SD rats were then randomly divided into 5 groups (n = 6 per group). To induce BPH, all rats, except for the control group, were given subcutaneous (S.C.) injections of TP (5 mg/kg) for 4 weeks. To minimize the animal suffering and distress during injections, these were carried out into the loose skin on the back of the neck and the site of injection varied to reduce local skin reactions. In addition, based on *in vitro* studies, appropriate drugs (finasteride, saw palmetto, SC extract) were administered orally for 4 weeks as follows: control group (PBS, P.O.), BPH group (testosterone/TP 5 mg/kg, S.C. + PBS, P.O.), Fina (TP 5 mg/kg, S.C. + Finasteride 10 mg/kg, P.O.), Saw (TP 5 mg/kg, S.C. + saw palmetto extract 100 mg/kg, P.O.), SC 50 (TP 5 mg/kg, S.C. + SC extract 50 mg/kg, P.O.). At the end of the experimental period, rats were fasted overnight and euthanized by CO_2_ asphyxiation in the euthanasia apparatus. Animals were left in the chamber for approximately 5 min until the visible movements ceased and euthanization was confirmed. Blood was drawn from the abdominal vein. Then, the blood samples were centrifuged at 3000 rpm at 4 ^o^C for 15 min, and the serum was stored at -80 ^o^C until further analysis. Serum testosterone and DHT levels were determined using a commercial enzyme-linked immunosorbent assay (ELISA) kit (ALPCO Diagnostics, NH, USA), according to the manufacturer’s protocol. Dissected prostate glands were weighed and stored in -80 ^o^C liquid nitrogen for further analysis. The body weight of rats was measured at the beginning and the end of the experimental period.

### Histological study

Paraffin-embedded prostate tissues were cut into 5 μm sections. After deparaffinization and dehydration, sections were subjected to hematoxylin and eosin (H&E) staining and examined using a light microscope (Nikon eclipse 80i, Nikon Corporation, Tokyo, Japan) at a × 400 magnification. Images were taken from 10 randomly selected fields, and epithelial thickness and lumen area were measured using Image J software (Image J v46a; NIH, USA).

### Western blot analysis

Both *in vitro* and *in vivo* western blotting were performed to assess BPH-related protein expression. In the *in vitro* study, LNCaP cells were seeded onto 6-well plates (5×10^5^ cells/well) in RPMI medium. One day later, cells were cultures in a medium containing 1 μM testosterone (Tokyo Chemical Ins. Co., Tokyo, Japan), except for the control group. After treatment with the appropriate drugs, finasteride (10 μM, Sigma, USA), saw palmetto extract (100 μg/mL), or SC extract (25, 50 μg/mL; the concentrations which showed high cell viability). After 72 h of treatment, cells were harvested for the preparation of total protein. The collected cells and frozen prostate tissue samples were lysed with RIPA buffer, centrifuged at 12,000 rpm for 20 min at 4 ^o^C, and the supernatant was used for western blotting. After protein quantification, samples were separated on 6–12% SDS-polyacrylamide gels and transferred onto a polyvinylidene fluoride (PVDF) membrane using a semi-dry transfer system (Bio-Rad, Hercules, CA, USA) After transfer, the membrane was rinsed with distilled water (dH_2_O) and stained with ponceau to confirm the efficiency of protein transfer and the membrane was blocked using a blocking buffer solution (5% non-fat dry milk in PBST) for 2 ½ h at room temperature (25 ^o^C). Then, membranes were incubated with various primary antibodies, as follows: anti-androgen receptor (AR, sc-7305, Lot # G0617, 1:1000, Santa Cruz, Texas, USA), anti-prostate specific antigen (PSA, sc-7316, Lot # A2717, 1:1000, Santa Cruz), anti-B-cell lymphoma 2 (Bcl-2, sc-7382, Lot # B0817, 1:1000, Santa Cruz), anti-B-cell lymphoma-associated X (Bax, sc-7480, Lot # I1217,1:1000, Santa Cruz), and anti-β-actin (ab8227, Lot: GR218829-1, 1:5000, Abcam, Cambridge, UK). Subsequently, membranes were washed with phosphate buffered saline Tween (PBST) and incubated with the secondary antibody, horseradish peroxidase-conjugated goat anti-rabbit (LF-SA8002, Lot. AT2718-NA09C, 1:5000; AbFrontier, Seoul, Korea). After washing with PBST, proteins were visualized using the enhanced chemiluminescence detection (ECL) kit (Amersham Pharmacia Biotech, Buckinghamshire, UK), and quantified using Image Lab Software (Bio-Rad, Hercules, CA, USA).

### Immunohistochemical (IHC) staining

For the IHC study, prostate tissues were fixed immediately in a 10% buffered formalin phosphate solution, embedded in paraffin (SAKURA Tissue-Tek TEC 5 Tissue Embedding Center, Japan), and cut into 5-μm sections (Leica RM 2135 microtome, Germany). After deparaffinization and dehydration, sections were subjected to 3% quenched endogenous H_2_O_2_ (in methanol), and treated with a 0.5% Triton X-100 solution for 30 min at room temperature (25 ^o^C). Then, nonspecific binding sites were blocked with normal goat serum (diluted 1:10 in PBS), and incubated overnight with primary antibodies, as follows: anti-5α-reductase type 2 (sc-20659, Lot # D2814, 1:100; Santa Cruz, Texas, USA) and anti- proliferating cell nuclear antigen (PCNA) (ab29, Lot: GR3195972-6, 1:3000, Abcam, MA, USA) at 4 ^o^C, incubated with the secondary antibody goat anti-rabbit (LF-SA8002, Lot. AT2718-NA09C, 1: 200, Ab Frontier, Seoul, Korea), and developed using a diaminobenzidine peroxidase substrate kit (Vector Laboratories, Burlingame, USA). Stained sections were examined for 5-α reductase type 2 and proliferating cell nuclear antigen (PCNA)-positive nuclei using a light microscope (Nikon eclipse 80i, Nikon Corporation, Tokyo, Japan) at × 200 and 400 magnifications, from 10 randomly selected fields of prostate tissues.

### Statistical analysis

All data are expressed as mean ± standard deviation (SD). Statistical analysis was performed with one-way ANOVA, with Tukey’s multiple comparison test for all pairwise comparisons of the mean value of different treatment groups using SigmaPlot 12 program. All the experimental data related to the SC extract-treated groups were compared with the normal control and the BPH groups. *P-values* < 0.05 were considered statistically significant. Graphs were constructed using GraphPad Prism 5.

## Results

### HPLC analysis of hederacoside D and morroniside in the SC extract

First, we identified the phytochemical compounds in the SC extract using HPLC. Hederacoside D is a major bioactive saponin that plays a pivotal role in overall biological activity. Morroniside is also an active phytochemical component. Hederacoside D showed a characteristic peak at 39.52 min, while morroniside, at 6.53 min. The concentrations of Hederacoside D and morroniside were 25 and 1.5 mg/g, respectively (S1 Fig).

### Cell viability and BPH-related protein expression

**Fig 1:**
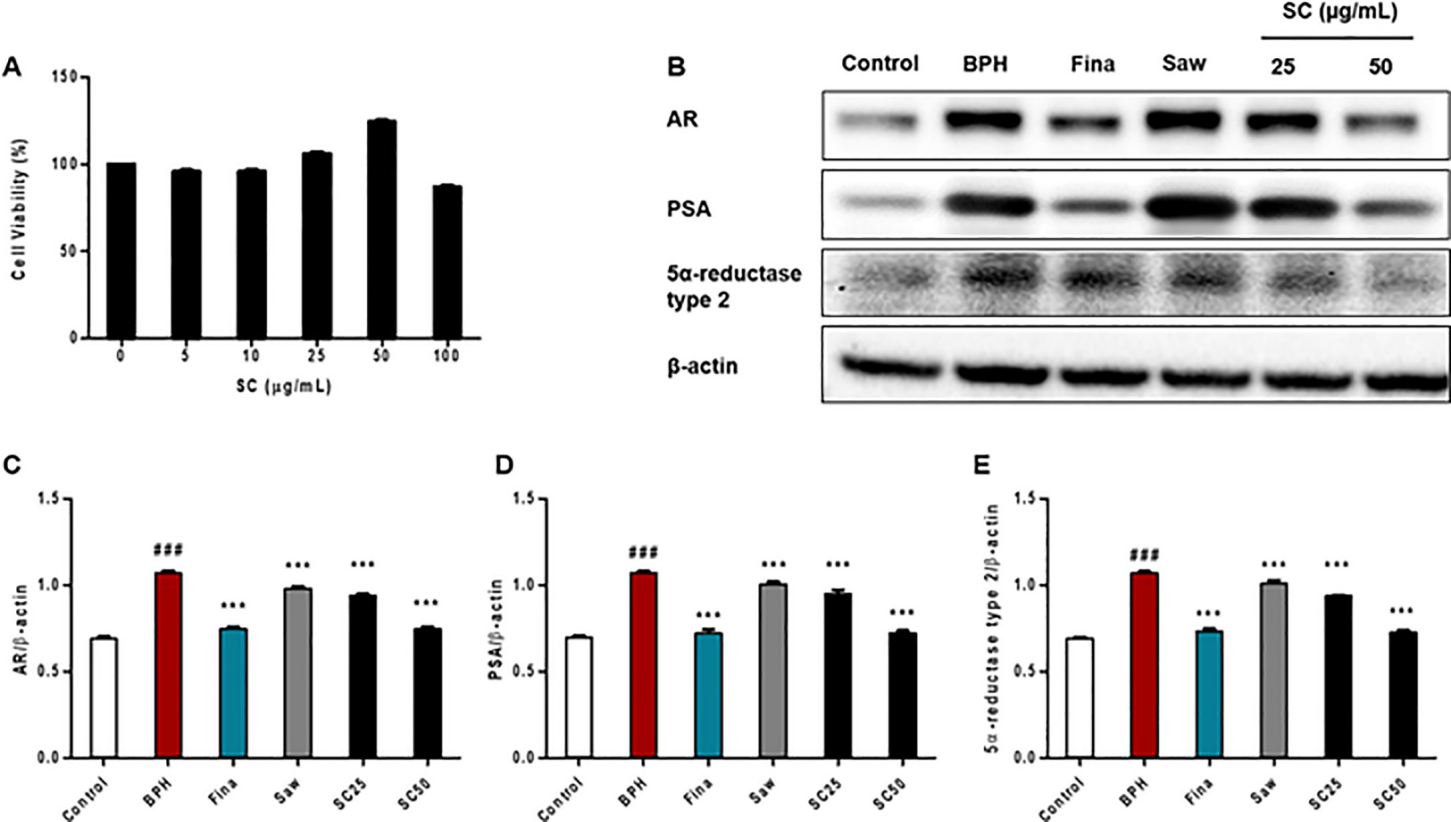
Effects of SC extract on the LNCaP cell line. LNCaP cells were treated with SC extract (0–100 μg/mL) for 72 h, and cell viability was determined by MTT assay, using EZ-Cytox Cell Viability assay kit. (A) Cell viability of SC extract. LNCaP cells were incubated in medium containing testosterone (1 μM), Finasteride (10 μM), Saw palmetto (100 μg/mL) or SC (25, 50 μg/mL) for 72 h. Then, cells were harvested and subjected to the western blot assay. (B) Representative western blot analysis of AR, PSA, and 5α-reductase type 2 in TP-induced BPH *in vitro*. (C, D, E) Relative densitometry evolution of AR, PSA, and 5α-reductase type 2, the western blot data respectively. Data are expressed as the means ± SD of three replicate experiments. ### *p* < 0.001 versus control group; *** *p* < 0.001 versus BPH group.

To examine the cytotoxicity of SC extract, an MTT assay was performed in LNCaP cells. As shown in Fig 1A, there was no significant difference in cell viability between SC-treated cells and control cells. However, exposure to 100 μg/mL SC extract markedly decreased cell viability by up to 86.73%, demonstrating that a high concentration inhibits cell viability due to its toxicity. The highest cell viability was observed in cells treated with 50 μg/mL. We then investigated the effect of SC extract on AR, PSA, and 5α-reductase type 2 protein expression in TP-induced LNCaP cells by using western blotting (Fig 1B). As shown in Fig 1C–1E, TP treatment significantly (*p <* 0.001) increased the expression of AR, PSA, and 5α-reductase type 2, relative to the control group. In contrast, the SC treated group showed a significantly (*p <* 0.001) decreased expression of AR, PSA, and 5α-reductase type 2, along with the Fina and Saw groups, compared to the BPH group. These results suggest that SC extract acts as a 5ARI, thereby suppressing androgen signaling in LNCaP cells.

### Effect of SC extract on body and prostate weight

Based on the *in vitro* results, we performed animal experiments to evaluate the efficacy of SC extract as an anti-BPH treatment. Body weight (BW) and prostate weight (PW) are commonly used to evaluate the progression of experimentally induced BPH *in vivo*. As shown in Table 1, the BPH group showed significantly (*p <* 0.001) increased PW and relative prostatic weight compared to the control group, while the Fina and SC 50 groups exhibited significantly (*p <* 0.001) decreased expression of the aforementioned parameters, compared with the BPH group. However, no significant differences in BW were observed among the groups.

**Table 1:** Effect of SC extract on prostate and body weight in TP-induced BPH rats.

	Body weight (g)		Prostate weight (g)	
	Initial	Final	Absolute	Relative
**Control**	250.9 ± 9.51	378.9 ± 40.92	0.66 ± 0.08	1.00 ± 0.18
**BPH**	230.9 ± 8.90	340.6 ± 23.12	1.25 ± 0.12^###^	2.07 ± 0.23^###^
**Fina**	230.7 ± 8.22	348.0 ± 23.15	1.01 ± 0.06***	1.64 ± 0.22***
**Saw**	230.8 ± 8.09	336.2 ± 22.75	1.24 ± 0.08	2.09 ± 0.17
**SC 50**	230.8 ± 7.73	346.8 ± 23.29	0.99 ± 0.14***	1.61 ± 0.21***

Abbreviations: Control (PBS, P.O.), BPH (5 mg/kg TP, S.C. + PBS, P.O.), Fina (5 mg/kg TP, S.C. + finasteride 10 mg/kg, P.O.), Saw (5 mg/kg TP, S.C. + saw palmetto 100 mg/kg, P.O.), SC 50 (5 mg/kg TP, S.C. + SC extract 50 mg/kg, P.O.). To induce BPH, all rats were received daily TP injection (5 mg/kg, S.C.) for consecutive 4 weeks, except for the control group. Data are expressed as the means ± SD (n = 6).

### *p* < 0.001 versus control group.

*** *p* < 0.001 versus BPH group.

### Morphological changes in prostate tissue in TP-induced BPH rats

**Fig 2:**
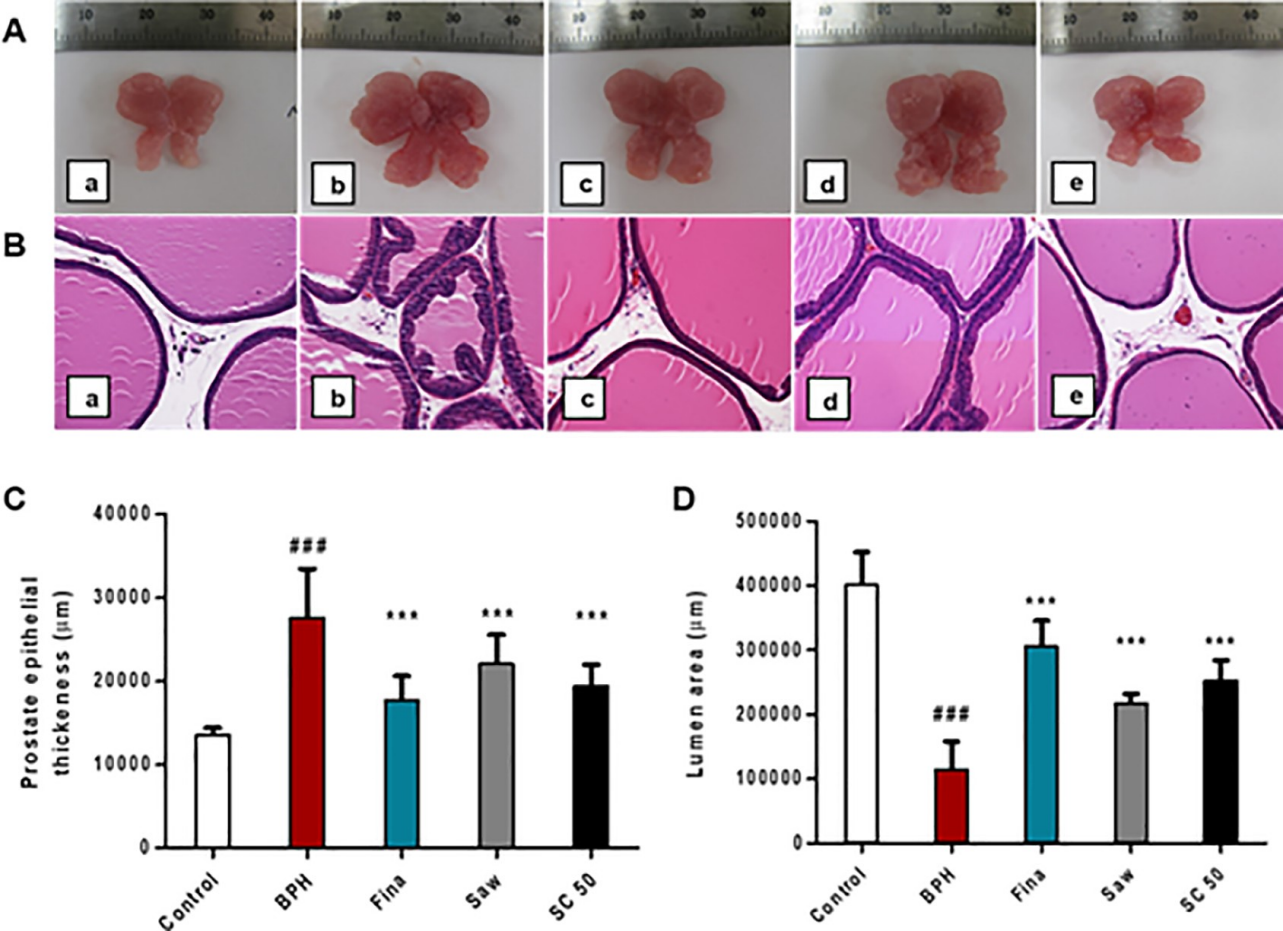
Effects of SC extract on prostate size and histopathology in TP-induced BPH rats. To induce BPH, all rats were daily received TP injection (5 mg/kg, S.C.), except for the control group along with appropriate drugs (finasteride, saw palmetto, SC extract) orally for 4 weeks. For histological studies, after deparaffinization and dehydration, prostate tissues were subjected to H&E staining. Then, stained sections were examined using a light microscope (magnification, ×400). (A) Comparison of the size of prostate gland. (B) Representative photomicrographs of prostate sections. (C) Measurement of the prostate epithelial thickness. (D) Measurement of the lumen area of the prostate tissues. (a) Control, (b) BPH, (c) Fina, (d) Saw, (e) SC 50. Abbreviations: Control (PBS, P.O.), BPH (5 mg/kg TP, S.C. + PBS, P.O.), Fina (5 mg/kg TP, S.C. + finasteride 10 mg/kg, P.O.), Saw (5 mg/kg TP, S.C. + saw palmetto 100 mg/kg, P.O.), SC 50 (5 mg/kg TP, S.C. + SC extract 50 mg/kg, P.O.). Data are expressed as the means ± SD (n = 6). ### *p* < 0.001 versus control group; *** *p* < 0.001 versus BPH group.

As shown in Fig 2A, the BPH group exhibited a markedly increased prostate size along with the Saw group relative to the control group; however, SC treatment markedly reduced the prostate size of TP-induced BPH rats, compared with the BPH group. Our histopathological examination (Fig 2B) demonstrated that the BPH group had a serious disruption in the histo-architecture of the prostate tissue, indicating stromal and epithelial proliferations, thereby glandular hyperplasia, hypertrophy, and significant thickening (*p <* 0.001; Fig 2C), with papillary projections in the epithelium. The lumen area of BPH group significantly (*p <* 0.001; Fig 2D) decreased, as compared to the control group, which was not associated with histological changes. However, SC treatment showed significant improvements in the histo-architecture of the prostatic tissue, such as a decreased prostate epithelial thickness (*p <* 0.001) and an increased lumen area (*p <* 0.001), along with the Fina and Saw groups, compared to the BPH group. These results indicate that SC extract could be an alternative treatment for prostatic hyperplasia by attenuating the abnormal morphological changes in BPH rats.

### SC mediated downregulation of serum androgens and BPH-related protein expression in TP-induced BPH rats

**Fig 3:**
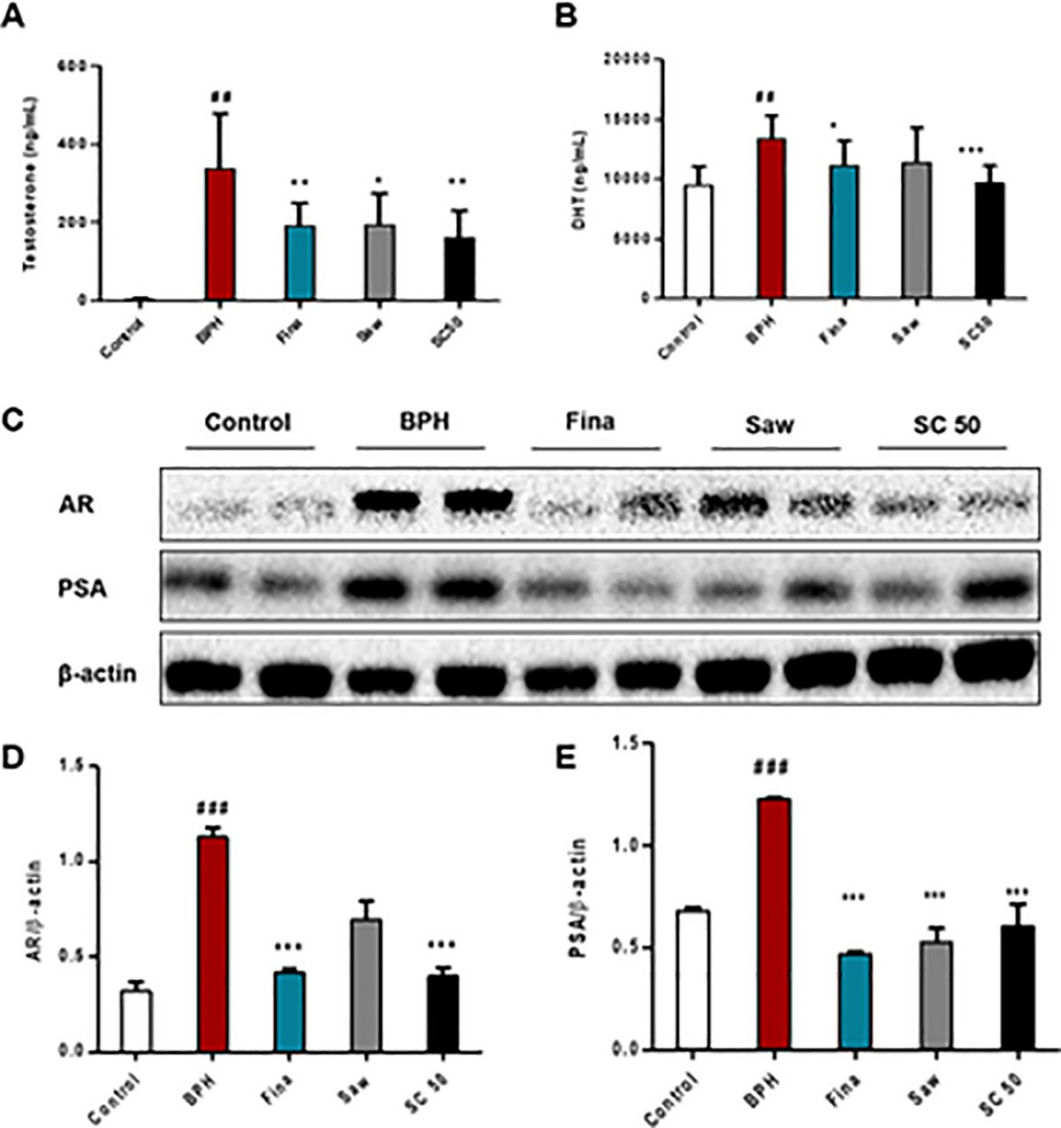
Effects of SC extract on serum androgens and BPH-related proteins expression in TP-induced BPH rats. To induce BPH, all rats were daily received TP injection (5 mg/kg, S.C.), except for the control group along with appropriate drugs (finasteride, saw palmetto, SC extract) orally for 4 weeks. Serum testosterone and DHT levels were evaluated using an ELISA assay. Western blotting was performed to assay AR and PSA, the BPH-related protein expressions. (A) The serum concentrations of testosterone. (B) The serum concentrations of DHT. (C) Western blot analysis of prostatic AR and PSA. (D, E) Relative densitometry evolution of AR and PSA, the western blot data. Abbreviations: Control (PBS, P.O.), BPH (5 mg/kg TP, S.C. + PBS, P.O.), Fina (5 mg/kg TP, S.C. + finasteride 10 mg/kg, P.O.), Saw (5 mg/kg TP, S.C. + saw palmetto 100 mg/kg, P.O.), SC 50 (5 mg/kg TP, S.C. + SC extract 50 mg/kg, P.O.). Data are expressed as the means ± SD (n = 6). ## *p* < 0.01 versus control group; ### *p* < 0.001 versus control group * *p* < 0.05 versus BPH group; ** *p* < 0.01 versus BPH group; *** *p* < 0.001 versus BPH group.

**Fig 4:**
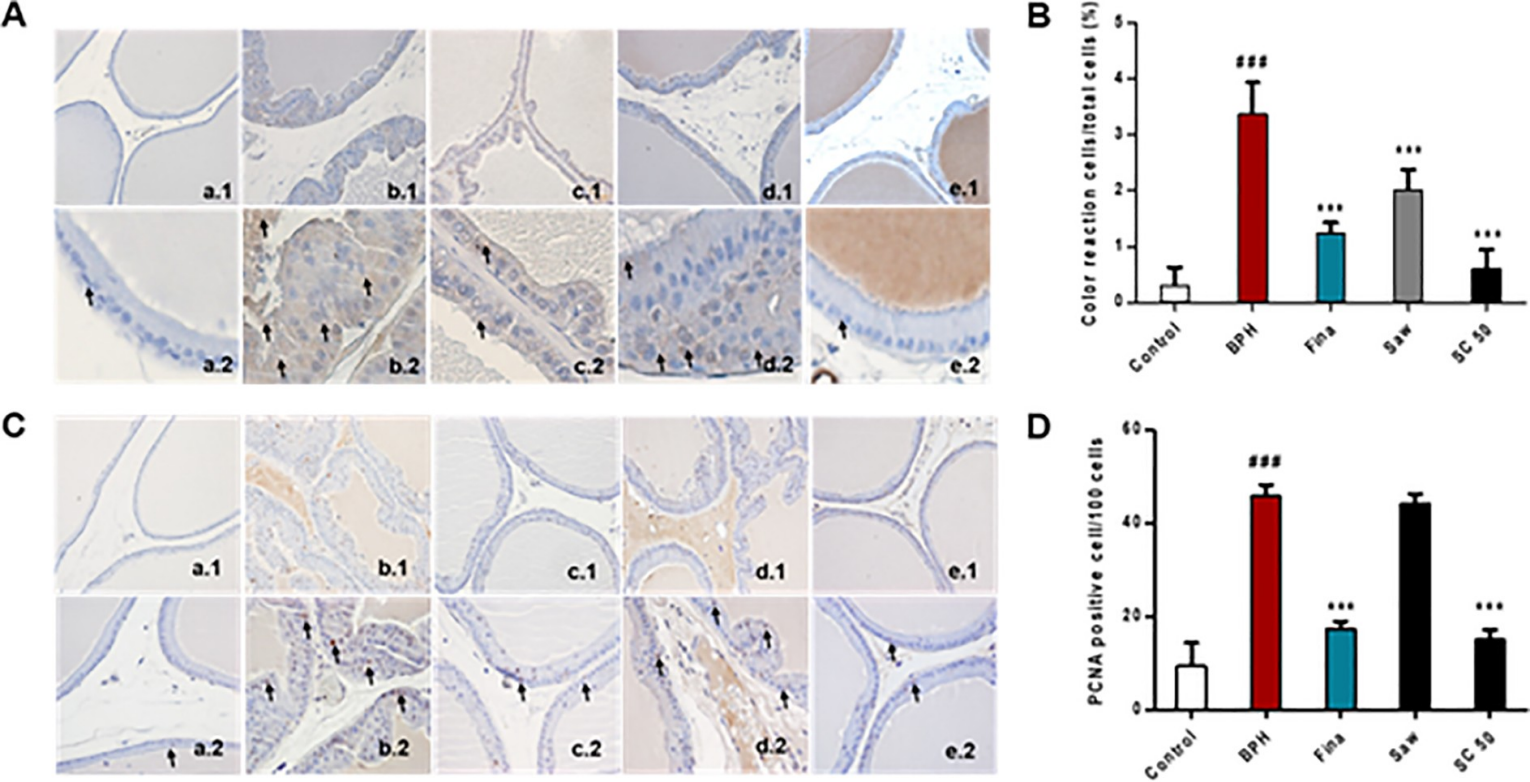
Effects of SC extract on 5α-reductase type 2 and prostate cell proliferation in TP-induced BPH rats. To induce BPH, all rats were daily received TP injection (5 mg/kg, S.C.), except for the control group along with appropriate drugs (finasteride, saw palmetto, SC extract) orally for 4 weeks. For immunohistochemistry assay, after deparaffinization and dehydration, prostate tissues were subjected to anti-5α-reductase type 2 and anti-PCNA, the primary antibodies followed by the secondary antibody, goat anti-rabbit. Then, stained sections were examined using a light microscope to analyze the 5α-reductase type 2 color reaction cells and PCNA positive nuclei in 10 randomly selected fields (magnifications: 1, ×200; 2, ×400; short arrows, 5α-reductase type 2 color reaction cells and PCNA positive cells). (A) Representative photomicrographs of 5α-reductase type 2 expression. (B) 5α-reductase type 2 color reaction cell (%) in the prostate epithelial cells. (C) Representative photomicrographs of PCNA expression. (D) PCNA positive cell/100 cells. (a) Control, (b) BPH, (c) Fina, (d) Saw, (e) SC 50. Abbreviations: Control (PBS, P.O.), BPH (5 mg/kg TP, S.C. + PBS, P.O.), Fina (5 mg/kg TP, S.C. + finasteride 10 mg/kg, P.O.), Saw (5 mg/kg TP, S.C. + saw palmetto 100 mg/kg, P.O.), SC 50 (5 mg/kg TP, S.C. + SC extract 50 mg/kg, P.O.). Data are expressed as the means ± SD (n = 6). ### *p* < 0.001 versus control group; *** p < 0.001 versus BPH group.

Next, we studied the expression of testosterone and DHT, which are factors in the progression of BPH in a TP-induced BPH model. As shown in Fig 3A, a significant (*p <* 0.01) increase in serum testosterone levels was observed in the BPH group compared with the control group. In contrast, the Fina, SC 50 (*p <* 0.01), and Saw (*p <* 0.05) groups showed significantly decreased testosterone levels relative to the BPH group. Similarly, serum DHT levels also significantly (*p <* 0.01) increased in the BPH group (Fig 2B) compared to the control, while SC treatment showed significantly (*p <* 0.001) decreased DHT levels, together with the Fina group (*p <* 0.05), relative to the BPH group. These results proved that SC extract is a potential treatment for reducing androgen concentrations in BPH rats. Then, we examined the expression of AR and PSA and BPH-related protein expression using western blotting (Fig 3C–3E) and BPH group showed a significantly (*p <* 0.001) increased expression of AR and PSA, compared with the control group. In contrast, as expected, the rats treated with finasteride and SC extract showed a significantly (*p <* 0.001) decreased expression of AR and PSA relative to the BPH group. In addition, the Saw group exhibited markedly decreased AR and significantly (*p <* 0.001) decreased PSA expression relative to the BPH group. To further confirm this result, immunostaining for 5α-reductase type 2 was carried out as it is the enzyme that catalyzes the conversion of testosterone into DHT, thereby causing abnormal growth of the prostate gland. As shown in Fig 4A and 4B, rats in the control group showed no abnormalities; however, those in the BPH group showed a significantly (*p <* 0.001) increased expression of 5α-reductase type 2, as indicated by the color reaction in the prostate epithelial cells (Fig 4A. b2: short arrows, color reaction in prostate epithelial cells). Notably, the SC extract along with finasteride and saw palmetto significantly (*p <* 0.001) reversed the effect of TP, compared with the BPH group. Together, these results indicate that SC extract has the ability to suppress androgen signaling in prostate cells through its 5α-reductase type 2 inhibitory effect and possible treatment for TP-induced BPH rats.

### SC-mediated regulation of apoptosis and cell proliferation in TP-induced BPH rats

**Fig 5:**
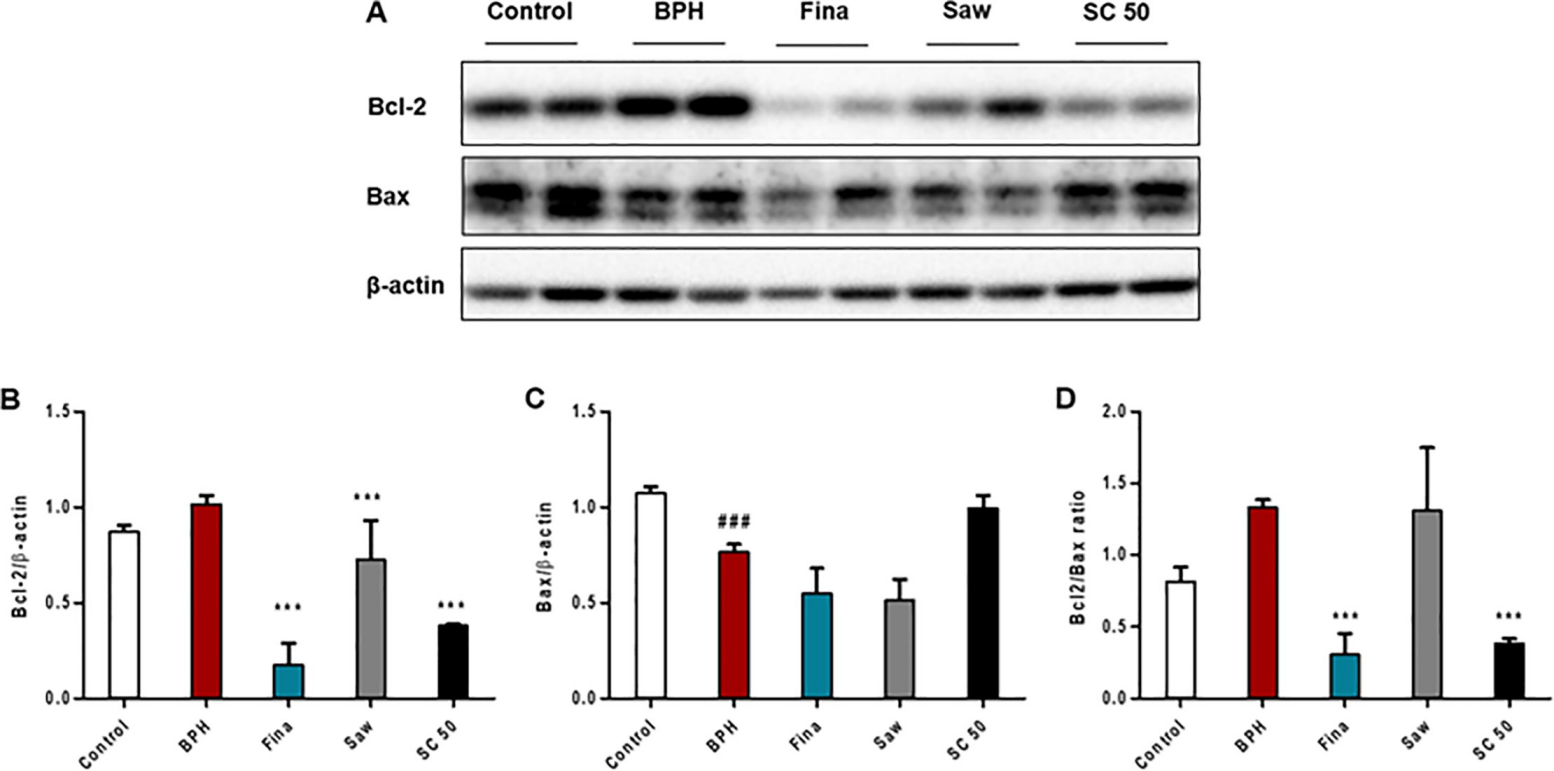
Effects of SC extract on the expression of Bcl-2 family proteins in prostate tissues in TP-induced BPH rats. To induce BPH, all rats were daily received TP injection (5 mg/kg, S.C.), except for the control group along with appropriate drugs (finasteride, saw palmetto, SC extract) orally for 4 weeks. Western blotting was performed to assay Bcl-2 and Bax, the Bcl-2 family proteins. (A) Western blot analysis of prostatic Bcl-2 and Bax. (B, C) Relative densitometry evolution of Bcl-2 and Bax, the western blot data. (D) Bcl-2/Bax ratio, which is used as a rheostat for determining cell susceptibility to apoptosis. Abbreviations: Control (PBS, P.O.), BPH (5 mg/kg TP, S.C. + PBS, P.O.), Fina (5 mg/kg TP, S.C. + finasteride 10 mg/kg, P.O.), Saw (5 mg/kg TP, S.C. + saw palmetto 100 mg/kg, P.O.), SC 50 (5 mg/kg TP, S.C. + SC extract 50 mg/kg, P.O.). Data are expressed as the means ± SD (n = 6). ### *p* < 0.001 versus control group; *** *p* < 0.001 versus BPH group.

During the progression of BPH, cell proliferation may increase and PCNA is a hallmark of proliferating cells in the prostate [29]. As shown in Fig 4C, the control group had a low expression of PCNA; however, rats in the BPH group exhibited a significantly (*p <* 0.001) increased number of PCNA-positive cells, which was characterized by brown-stained nuclei (Fig 4C and 4D; short arrows, PCNA-positive cells). Finasteride and SC extract significantly (*p <* 0.001) reversed the effect of TP, as compared to the BPH group. Next, the expression of Bcl-2 and Bax was examined to study the effect of SC extract on apoptosis of BPH *in vivo* (Fig 5A). As shown in Fig 5B and 5C, the expression of Bcl-2, which includes the anti-apoptotic factor upregulated and the pro-apoptotic factor Bax significantly (*p <* 0.001) downregulated in the BPH group, compared with the control group. SC extract significantly (*p <* 0.001) decreased the expression of Bcl-2, but increased the expression of Bax relative to the BPH group. In addition, both the Fina and Saw groups exhibited a significantly (*p <* 0.001) decreased expression of Bcl-2 compared to the BPH group. Further, the ratio of Bcl-2 to Bax significantly (*p <* 0.001) decreased in both the Fina and SC 50 groups, compared with the BPH group, where the ratio of Bcl-2 to Bax markedly increased compared to the control group (Fig 5D). These results proved that SC extract is a possible treatment for the modulation of PCNA and Bcl-2 family proteins and induces apoptosis in a positive manner. Therefore, SC extract is a potential treatment to maintain the balance between cell proliferation and apoptosis in TP-induced BPH.

## Discussion

Benign prostatic hyperplasia and associated LUTS are common urological problems among older men. Androgens, such as testosterone and DHT, play a fundamental role in the development and maintenance of BPH [15]. The activities of these androgens can be regulated by adding a 5ARI, such as finasteride. However, finasteride is a drug that has been associated with adverse effects, such as sexual dysfunction and allergic reactions. In addition, a previous clinical study has revealed that patients subjected to 5ARI treatment can be exposed to a higher risk of high-grade prostate cancer [30]. Therefore, to substitute for synthetic drugs and reduce side effects, this study investigated the therapeutic effects of plant-based medicine using SC extract on BPH *in vitro* and *in vivo*.

First, we conducted *in vitro* investigations to study the effect of SC extract on TP-induced BPH in the LNCaP cell line. During the progression of BPH, testosterone is converted into DHT by 5α-reductase type 2. DHT then binds to the nuclear ARs, and this complex gives rise to PSA, which is a good predictor of BPH progression [31]. Furthermore, this complex is involved in the prostatic expression of vascular endothelial growth factor, epidermal growth factor, and basic fibroblast growth factor, and finally promotes angiogenesis and prostatic growth [32]. Therefore, we first assayed the expression of AR, PSA, and 5α-reductase type 2 *in vitro*. According to our results, SC extract significantly reduced the expression of AR, PSA, and type 2 5α-reductase. These findings suggest that SC extract acts as a 5ARI, thereby suppressing androgen signaling in LNCaP cells and a possible treatment for TP-induced BPH. Therefore, we performed *in vivo* investigations to further examine the therapeutic effect of SC extract on the TP-induced BPH rat model.

Examination of BW, PW, and serum androgens is currently used to evaluate the progression of BPH. In line with previous studies [16, 33], our investigation also demonstrated that rats with experimentally induced BPH have higher PW and serum androgens, such as testosterone and DHT. Testosterone is an androgen produced by Leydig cells of the testis, while DHT is an important byproduct of testosterone and the most potent androgen in men, which is involved in development and aging. Our study demonstrated significantly increased testosterone and DHT levels in the BPH group. However, SC extract significantly reduced the expression of the aforementioned serum androgens. These data suggest that SC extract has the ability to regulate androgen and its byproducts, thereby attenuating BPH development and progression through its anti-androgenic activity.

BPH is a precancerous prostate condition, which is caused by the overgrowth of prostatic epithelial and stromal cells, and arises due to the imbalance between cell proliferation and apoptosis [34]. Hyperplasia of these cells results in increased prostate weight and causes contraction of the urethral canal, which may obstruct the urine flow [17]. Histologically, BPH is characterized by the proliferation of stromal and glandular cells [35]. Our histological examination demonstrated that the BPH group had a serious interruption in the histo-architecture of prostate tissue. However, SC extract significantly recovered these abnormal morphological changes, thereby reducing the prostate size of TP-induced BPH rats.

Androgens affect gene expression in various kinds of tissues and cells by binding with AR, which has been linked to prostate cancer [36, 37]. DHT has a higher affinity for AR than testosterone. In the prostate, the interaction between DHT and AR results in the production of proteins, such as PSA [38, 39]. PSA, which is a glycoprotein in humans, is encoded by the kallikrein-related peptidase 3 (KLK3) gene and is secreted by the prostatic epithelial cells. As serum PSA level is frequently elevated in prostate disorders such as BPH and prostate cancer, it is used as a clinical marker for disease prognosis [40]. In the current study, immunoblotting revealed that SC extract significantly reduced the expression of AR and markedly decreased PSA expression, indicating that SC extract has the ability to suppress androgen signaling in prostate cells of BPH rats. This result was further examined by immunostaining of 5α-reductase type 2, as it is the convertor of testosterone to its active form, DHT, and is involved in BPH development and progression [41]. As expected, SC extract reduced type-2 5α-reductase expression and, together, these results proved that the SC extract acts as a 5ARI.

Progression of BPH is associated with several molecular mechanisms. As described above, AR-related 5α-reductase type 2 activity is considered a fundamental pathway of BPH. In addition, the balance between cell proliferation and apoptosis also plays a pivotal role in the BPH condition. Hyperplasia of prostatic epithelial and stromal cells is the major pathological feature of BPH, which is caused by an imbalance between cell proliferation and cell death [42]. In this study, the processes of cell proliferation and programed cell death were comparatively analyzed to determine the underlying mechanism of pathogenesis of BPH by using PCNA and Bcl-2 family proteins. Cell proliferation is related to the progression of the cell cycle, which is composed of four phases: G1, S, G2, and M. These phases are controlled by cell-cycle regulatory proteins [43]. PCNA is a nuclear protein that is elevated during the G1/S phase transition, thereby acting as a marker for proliferating cells and plays a crucial role in physiological conditions such as BPH [43]. To investigate the anti-proliferative effect of the SC extract, PCNA-positive cells in both glandular epithelium and stroma cells were examined. Our IHC results of PCNA demonstrated that SC extract significantly downregulated the expression of PCNA-positive cells, which is characterized by brown-stained nuclei. The Bcl-2 family has been identified for its role in apoptosis due to its Bcl-2 homology domains and involvement in the regulation of apoptosis. These Bcl-2 homology domains encourage family members interactions with each other. It also indicates pro- or anti-apoptotic functions. [44, 45]. The ratio of pro- to anti-apoptotic subfamily members in a cell may vary according to the number of signaling pathways and efficiency of transmitting information upon cellular stress, such as available nutrients, DNA damage, and protein processing [46]. Intrinsic apoptosis is regulated by anti-apoptotic Bcl-2, a protein localized in the mitochondrial outer membrane and suppresses the release of the pro-apoptotic factor cytochrome c [47]. The Bax gene is a pro-apoptotic protein of the Bcl-2 family and activates the apoptotic signal. The ratio of Bcl-2 to Bax determines the apoptotic reactivity [48]. Under normal conditions, prostate tissues exhibit relatively low levels of Bcl-2 expression, which are associated with low levels of apoptosis, while in BPH, the incidence of imbalance between apoptosis and proliferation is indicated by either apoptosis signals or by increasing PCNA expression [49]. In this study, SC extract significantly downregulated the anti-apoptotic Bcl-2 and markedly increased the level of pro-apoptotic Bax. Moreover, the Bcl-2/Bax ratio was significantly decreased by using SC extract treatment. Collectively, these findings suggest that SC extract is a possible treatment for inhibiting cell proliferative activity and inducing apoptosis in TP-induced BPH rat models.

## Conclusions

In conclusion, the findings of our study revealed that SC treatment significantly reduced prostate hyperplasia and prostate size, causing marked degenerative changes in prostatic stromal and epithelial cells. SC extract also prevents the 5α-reductase type 2-AR pathway by acting as a 5ARI, thereby reducing the conversion of testosterone into DHT. Furthermore, SC extract downregulated cell proliferation-related protein expression and induced cell apoptosis under BPH conditions. Hence, SC extract is a potential phytotherapeutic agent for modulating the balance between cell proliferation and apoptosis, which underlies the pathogenesis of BPH. Therefore, this investigation demonstrated that the combination of *S*. *hexaphylla* and *C*. *officinalis* has a protective effect against testosterone-induced benign prostatic hyperplasia through inhibition of 5α-reductase type 2 and induced apoptosis. The use of SC extract as a therapeutic intervention for the treatment of BPH warrants further investigation.

## Supporting information

S1 Fig HPLC chromatogram of hederacoside D and morroniside in SC extract. (A) Molecular structure of hederacoside D. (B) Constituent of hederacoside D in the SC extract analyzed by HPLC-PDA. (C) Molecular structure of morroniside. (D) Constituent of morroniside in the SC extract analyzed by HPLC-PDA.
(PPTX)

S1 Raw images (PPTX)

## Abbreviations

BPH: benign prostatic hyperplasia
LUTS: lower urinary tract symptoms
LNCaP: human prostate adenocarcinoma cells
TP: testosterone propionate
DHT: dihydrotestosterone
5ARIs: 5-α reductase type 2 inhibitors
AR: androgen receptor
PSA: prostate specific antigen
PCNA: proliferating cell nuclear antigen
Bcl-2: B-cell lymphoma 2
Bax: B-cell lymphoma-associated X
HPLC: high performance liquid chromatography
SD: Sprague-Dawley
S.C.: subcutaneous
P.O.: Per os/oral administration
IHC: immunohistochemistry
KLK3: kallikrein-related peptidase 3

## References

[1] Wang HB, Mayer R, Rocker G, Yang JJ, Matteson DS. A phenolic glycoside and triterpenoids from *stauntonia hexaphylla*. Phytochemistry. 1998;47: 467–470. 10.1016/S00319422(97)00588-8

[2] Cheon YH, Baek JM, Park SH, Anh SJ, Lee MS. *Stauntonia hexaphylla* (Lardizabalaceae) leaf methanol extract inhibits osteoclastogenesis and bone resorption activity via proteasome-mediated degradation of c-Fos protein and suppression of NFATc1 expression. BMC Compliment Altern Med. 2015;15 10.1186/s12906-015-0801-6 26271279 PMC4535770

[3] Marino G, Niso-Santano M, Baehrecke EH, Kroemer G. Self-consumption: the interplay of autophagy and apoptosis. Nat. Rev. Mol. Cell Biol. 2014;15: 81–94. 10.1038/nrm3735 24401948 PMC3970201

[4] Hwang SH, Kwon SH, Kim SB, Lim SS. Inhibitory activities of *Stauntonia hexaphylla* leaf constituents on rat lens aldose reductase and formation of advanced glycation end products and antioxidant. Biomed. Res. 2017:4273257 10.1155/2017/4273257 28326319 PMC5343222

[5] Vinh LB, Jang HJ, Phong NV, Cho KW, Park SS, Kang JS, et al Isolation, structural elucidation, and insights into the anti-inflammatory effects of triterpene saponins from the leaves of *stauntonia hexaphylla*. Bioorg. Med. Chem. Lett. 2019;29: 965–969. 10.1016/j.bmcl.2019.02.022 30808589

[6] Vinh LB, Jang HJ, Phong NV, Dan G, Cho KW, Kim YH, et al Bioactive triterpene glycosides from the fruit of *Stauntonia hexaphylla* and insights into the molecular mechanism of its inflammatory effects. Bioorg. Med. Chem. Lett. 2019;29: 2085–2089. 10.1016/j.bmcl.2019.07.010 31301930

[7] Dong Y, Feng ZL, Chen HB, Wang FS, Lu JH. *Corni Fructus*: a review of chemical constituents and pharmacological activities. Chinese Medicine. 2018;13 10.1186/s13020-018-0191-z PMC6020197 29983732

[8] Czerwinska ME and Melzig MF. Cornus mas and *Cornus officinalis*- Analogies and Differences of Two Medicinal Plants Traditionally Used. Front Pharmacol. 2018;9 10.3389/fphar.2018.00894 PMC6121078 30210335

[9] Hwangbo H, Kwon DH, Choi EO, Kim MY, Ahn KI, Ji SY. Corni Fructus attenuates testosterone-induced benign prostatic hyperplasia by suppressing 5α-reductase and androgen receptor expression in rats. Nutr Res Pract. 2018;12: 378–386. 10.4162/nrp.2018.12.5.378 30323905 PMC6172175

[10] Hong GL, Park SR, Jung DY, Karunasagara S, Lee KP, Koh EJ, et al The therapeutic effects of *Stauntonia hexaphylla* in benign prostate hyperplasia are mediated by the regulation of androgen receptors and 5α-reductase type 2. J Ethnopharmacol. 2020;250: 112446 10.1016/j.jep.2019.112446 31812646

[11] Untergasser G, Madersbacher S, Berger P. Benign prostatic hyperplasia: age-related tissue remodeling. Exp Gerontol. 2005;40: 121–128. 10.1016/j.exger.2004.12.008 15763388

[12] Lepor H. Pathophysiology of lower urinary tract symptoms in the aging male population. Rev Urol. 2005;7: S3˗S11. 16986059 PMC1477625

[13] Rosen R, Altwein J, Boyle P, Kirby RS, Lukacs B, Meuleman E, et al Lower urinary tract symptoms and male sexual dysfunction: the multinational survey of the aging male (MSAM-7). Eur. Urol. 2003;44: 637–649. 10.1016/j.eururo.2003.08.015 14644114

[14] Speakman M, Kirby R, Doyle S, Ioannou C. Burden of male lower urinary tract symptoms (LUTS) suggestive of benign prostatic hyperplasia (BPH)—focus on the UK. BJU Int. 2015;115: 508–519. 10.1111/bju.12745 24656222

[15] Nicholson TM and Ricke WA. Androgens and estrogens in benign prostatic hyperplasia: past, present and future. Differentiation. 2011;82: 184˗199. 10.1016/j.diff.2011.04.006 21620560 PMC3179830

[16] Andriole G, Bruchovsky N, Chung LW, Matsumoto AM, Rittmaster R, Roehrborn C, et al Dihydrotestosterone and the prostate: the scientific rationale for 5alpha-reductase inhibitors in the treatment of benign prostatic hyperplasia. J Urol. 2004;172: 1399–1403. 10.1097/01.ju.0000139539.94828.29 15371854

[17] Shin IS, Lee MY, Ha HK, Seo CS, Shin HK. Ursolic acid reduces prostate size and dihydrotestosterone level in a rat model of benign prostatic hyperplasia. Food Chem. Toxicol. 2012;50: 884˗888. 10.1016/j.fct.2012.01.007 22266360

[18] Lepor G, Kazzazi A, Djavan B. Alpha-blockers for benign prostatic hyperplasia: the new era. Curr. Opin. Urol. 2012;22: 7–15. 10.1097/MOU.0b013e32834d9bfd 22080875

[19] Kappor A. Benign prostatic hyperplasia (BPH) management in the primary care setting, Can. J. Urol. 2012;19 Suppl. 1: 10–17. 23089343

[20] Gravas S and Oelke M. Current status of 5α-reductase inhibitors in the management of lower urinary tract symptoms and BPH. World J. Urol. 2010;28: 9˗15. 10.1007/s00345-009-0493-y 19956956 PMC2809314

[21] Suzuki M, Ito Y, Fujino T, Abe M, Umegaki K, Onoue S, et al Pharmacological effects of Saw palmetto extract in the lower urinary tract. Acta pharmacol sin. 2009;30: 271˗281. 10.1038/aps.2009.1 19262550 PMC4002402

[22] Fagelman E, Lowe FC. Saw Palmetto Berry as a Treatment for BPH. Rev Urol. 2001;3: 134–138. 16985705 PMC1476047

[23] Colado‐Velázquez J, Mailloux‐Salinas P, Medina‐Contreras JML, Cruz‐Robles D, Bravo G. Effect of *Serenoa Repens* on Oxidative Stress, Inflammatory and Growth Factors in Obese Wistar Rats with Benign Prostatic Hyperplasia. Phytother. Res. 2015; 29: 1525–1531. 10.1002/ptr.5406 26104840

[24] Habib FK. Serenoa repens: the scientific basis for the treatment of benign prostatic hyperplasia. Eur Urol Suppl. 2009; 8: 887–893. 10.1016/j.eursup.2009.11.005

[25] Bullock TL and Andriole GL. Emerging drug therapies for benign prostatic hyperplasia. Expert Opin Emerg Drugs. 2006;11: 111˗123. 10.1517/14728214.11.1.111 16503830

[26] Traish AM, Hassani J, Guay AT, Zitzmann M, Hansen ML. Adverse side effects of 5α-reductase inhibitors therapy: Persistent diminished libido and erectile dysfunction and depression in a subset of patients. J Sex Med. 2011;8: 872˗884. 10.1111/j.1743-6109.2010.02157.x 21176115

[27] Raft MC. Social controls on cell survival and cell death. Nature. 1992;256: 396–400. 10.1038/356397a0 1557121

[28] Vacherot F, Azzouz M, Gil-Diez-de-Medina S, Colombel M, Taille ADL, Lefre`re Belda MA, et al Induction of Apoptosis and Inhibition of Cell Proliferation by the Lipido-Sterolic Extract of *Serenoa repens* (LSESr, PermixonT) in Benign Prostatic Hyperplasia. The Prostate. 2000;45: 259–266. 10.1002/1097-0045(20001101)45:3&lt;259::aid-pros9&gt;3.0.co;2-g 11074529

[29] Jin BR, Kim HJ, Park SK, Kim MS, Lee KH, Yoon IJ et al Anti-Proliferative Effects of HBX-5 on Progression of Benign Prostatic Hyperplasia. Molecules. 2018;23: 2638 10.3390/molecules23102638 30322186 PMC6222778

[30] Thompson IM, Goodman PJ, Tangen CM, Lucia MS, Miller GJ, Ford LG, et al The influence of finasteride on the development of prostate cancer. N. Engl. J. Med. 2003;349: 215–224. 10.1056/NEJMoa030660 12824459

[31] Cynthia AH, Chawnshang C. Androgen receptor in prostate cancer. Endocr. Rev. 1997;25: 276–308. 10.1210/er.2002-0032 15082523

[32] Briganti A, Capitanio U, Suardi N, Gallina A, Salonia A, Bianchi M, et al Benign prostatic hyperplasia and its aetiologies. Eur. Urol. 2009; Suppl.8: 865–871. 10.1016/j.eursup.2009.11.00218922627

[33] Arruzazabala ML, Mas R, Molina V, Noa M. Effect of D-004, a lipid extract from the Cuban royal palm fruit, on atypical prostate hyperplasia induced by phenylephrine in rats. Drugs R D. 2006;7: 233˗241. 10.2165/00126839-200607040-00003 16784248

[34] Afriyie DK, Asare GA, Bugyei K, Adjei S, Lin J, Peng J, et al Treatment of benign prostatic hyperplasia with Croton Membranaceus in an experimental animal model. J Ethnopharmacol. 2014;157: 90˗98. 10.1016/j.jep.2014.09.007 25256687

[35] Lee CH, Akin-Olugbade O, Kirschenbaum A. Overview of prostate anatomy, histology, and pathology. Endocrinol. Metab. Clin. North Am. 2011;40: 565–575. 10.1016/j.ecl.2011.05.012 21889721

[36] Guo Z, Yang X, Sun F, Jiang R, Linn DE, Chen H, et al A novel androgen receptor splice variant is up-regulated during prostate cancer progression and promotes androgen depletion-resistant growth. Cancer Res. 2009;69: 2305–2313. 10.1158/0008-5472.CAN-08-3795 19244107 PMC2672822

[37] Wilson CM and McPhaul MJ. A and B forms of the androgen receptor are expressed in a variety of human tissues. Mol Cell Endocrinol. 1996;120: 51–57. 10.1016/0303-7207(96)03819-1 8809738

[38] Chatterifee B. The role of the androgen receptor in the development of prostatic hyperplasia and prostate cancer. Mol. Cell. Biochem. 2003;253: 89–101. 10.1023/a:1026057402945 14619959

[39] Izumi K, Mizokami A, Lin WJ, Lai KP, Chan C. Androgen receptor roles in the development of benign prostate hyperplasia. Am. J. Pathol. 2013;182: 1942–1949. 10.1016/j.ajpath.2013.02.028 23570837 PMC3668026

[40] Chang RT, Kirby R, Challacombe BJ. Is there a link between BPH and prostate cancer?, Practitioner. 2012;256: 13˗16. 22792684

[41] Heike W, Michael K. Kinetic analysis of androstenedione 5α-reductase in epithelium and stroma of human prostate. Steroids. 1997;62: 589–594. 10.1016/s0039-128x(97)00042-1 9432753

[42] Zachary JF, McGavin MD. Pathologic Basis of Veterinary Disease (5th ed). Elsevier; St.Louis: e9780323291729. Mo (2012)

[43] Schonenberger F, Deutzmann A, Ferrando-May E, Merhof D. Discrimination of cell cycle phases in PCNA-immunolabeled cells. BMC Bioinforma. 2015;16: 180 10.1186/s12859-015-0618-9 26022740 PMC4448323

[44] Petros AM, Medek A, Nettesheim DG, Kim DH, Yoon HS, Swift K, et al Solution structure of the antiapoptotic protein bcl-2. Proc. Natl Acad. Sci. USA. 2001;98: 3012–3017. 10.1073/pnas.041619798 . 11248023 PMC30598

[45] Yin XM, Oltvai ZN, Korsmeyer SJ. BH1 and BH2 domains of Bcl-2 are required for inhibition of apoptosis and heterodimerization with Bax. Nature. 1994;369: 321–323. 10.1038/369321a0 . 8183370

[46] Hetz C and Glimcher L. The daily job of night killers: alternative roles of the BCL-2 family in organelle physiology. Trends Cell Biol. 2008;18: 38–44. 10.1016/j.tcb.2007.10.003 . 18077169

[47] Xu D, Wang X, Jiang C, Ruan Y, Xia S, Wang X. The androgen receptor plays different roles in macrophage-induced proliferation in prostate stromal cells between transitional and peripheral zones of benign prostatic hypertrophy. Excli Journal. 2017;16: 939–948. 10.17179/excli2017-335 . 28694768 PMC5500834

[48] Salakou S, Kardamakis D, Tsamandasetal AC, Zolota V, Apostolakis E, Tzelepi V. Increased Bax/Bcl-2 ratio up-regulates caspase-3 and increases apoptosis in the thymus of patients with myasthenia gravis. In Vivo. 2007; 21: 123–132. 17354625

[49] McVary KT. BPH: Epidemiology and comorbidities. Am J Manag Care. 2006;12: S122–S128. . 16613526

