# Supplementary material for: Protective effects of combination of Stauntonia hexaphylla and Cornus officinalis on testosterone-induced benign prostatic hyperplasia through inhibition of 5α- reductase type 2 and induced cell apoptosis
Source: PLoS One. 2020 Aug 13;15(8):e0236879. doi: 10.1371/journal.pone.0236879 (PMC7425886; doi:10.1371/journal.pone.0236879)
Supplement: S1 Fig — (A) Molecular structure of hederacoside D. (B) Constituent of hederacoside D in the SC extract analyzed by HPLC-PDA. (C) Molecular structure of morroniside. (D) Constituent of morroniside in the SC extract analyzed by HPLC-PDA. (PPTX) [file pone.0236879.s001.pptx]

## Slide 1
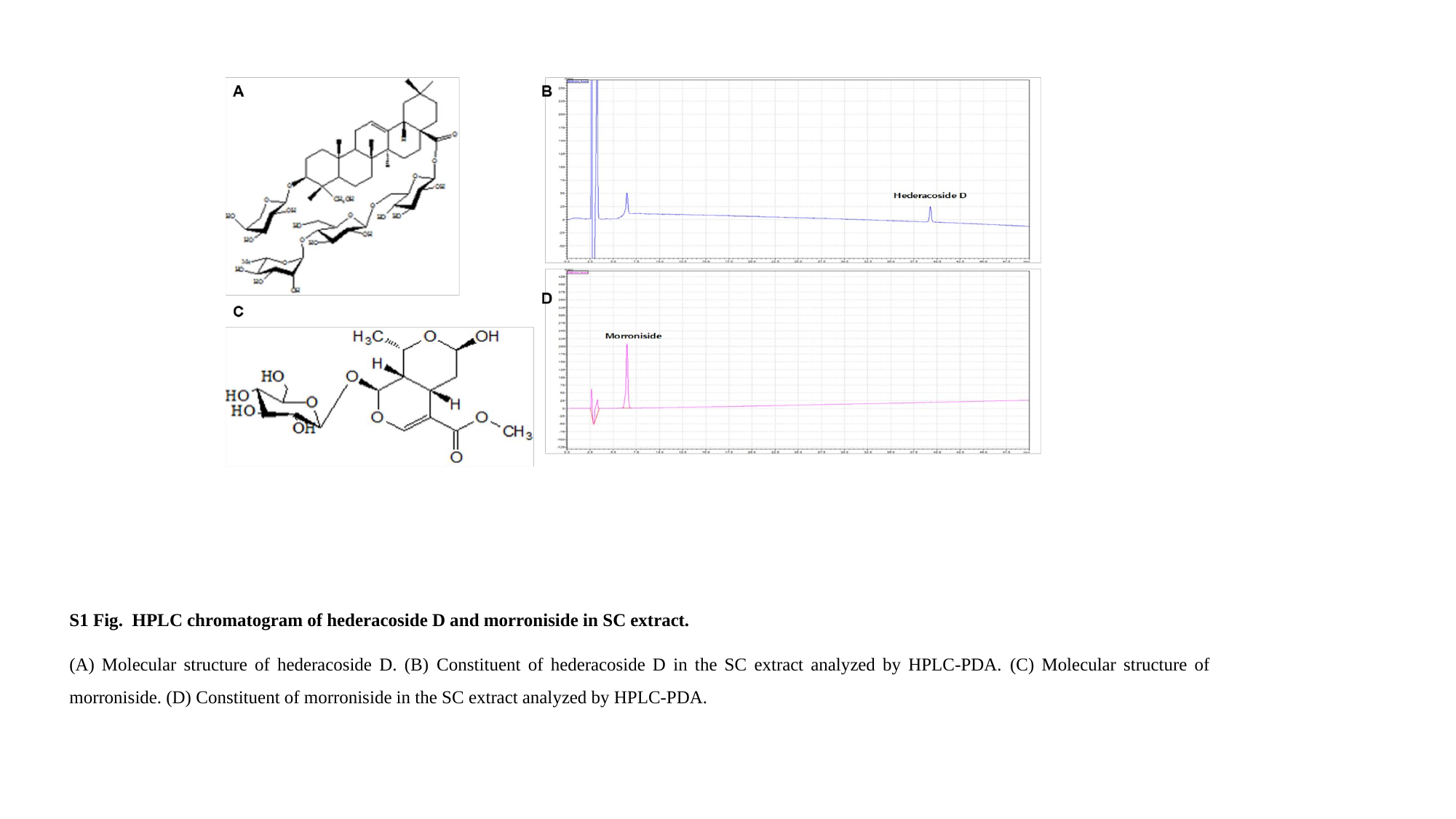

S1 Fig. HPLC chromatogram of hederacoside D and morroniside in SC extract.
(A) Molecular structure of hederacoside D. (B) Constituent of hederacoside D in the SC extract analyzed by HPLC-PDA. (C) Molecular structure of morroniside. (D) Constituent of morroniside in the SC extract analyzed by HPLC-PDA.
