# Supplementary material for: Protective effects of combination of Stauntonia hexaphylla and Cornus officinalis on testosterone-induced benign prostatic hyperplasia through inhibition of 5α- reductase type 2 and induced cell apoptosis
Source: PLoS One. 2020 Aug 13;15(8):e0236879. doi: 10.1371/journal.pone.0236879 (PMC7425886; doi:10.1371/journal.pone.0236879)
Supplement: S1 Raw images — (PPTX) [file pone.0236879.s002.pptx]

## Slide 1
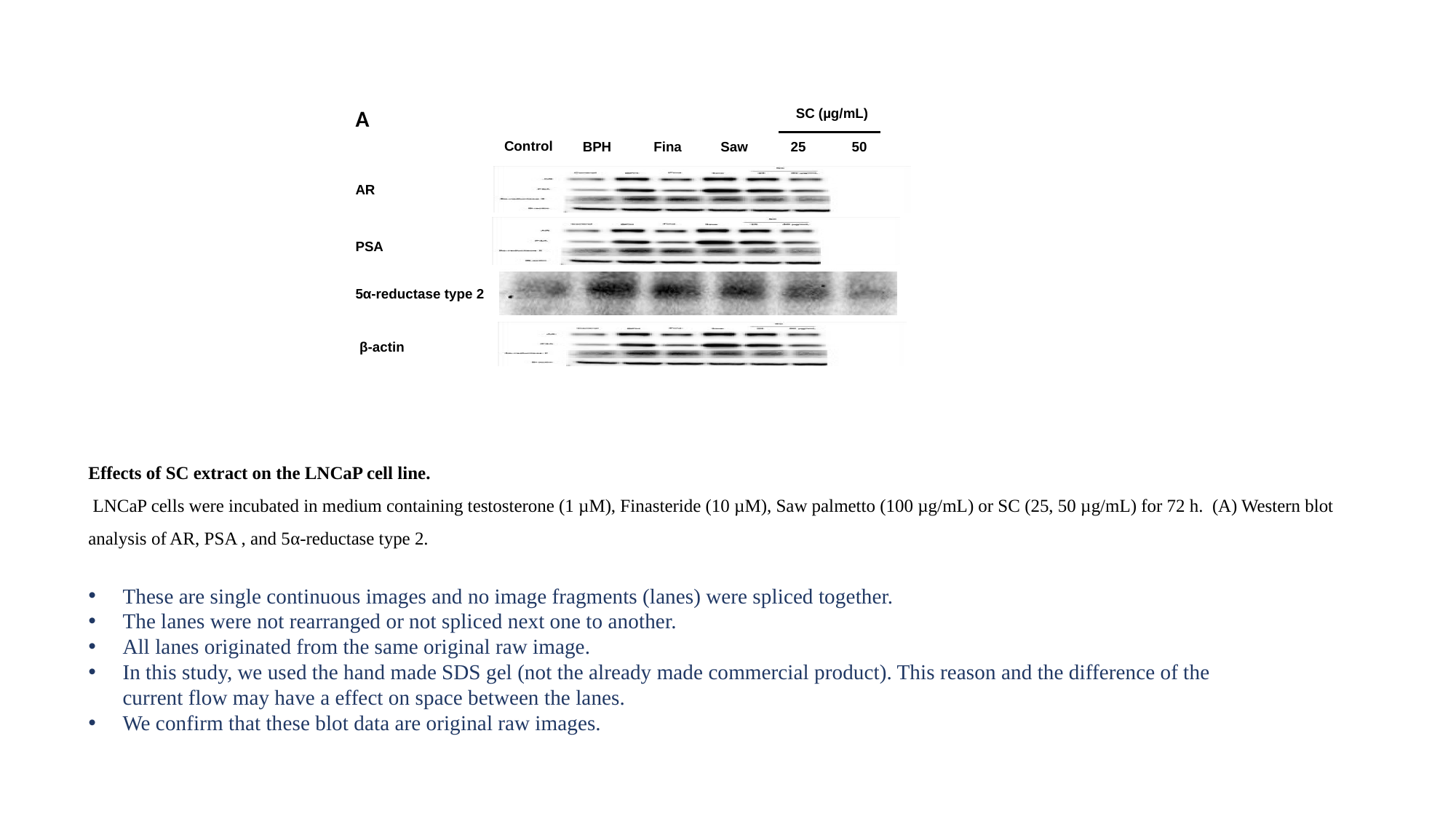

A
SC (µg/mL)
Control
BPH
Fina
 25 50
Saw
AR
PSA
5α-reductase type 2
β-actin
Effects of SC extract on the LNCaP cell line.
 LNCaP cells were incubated in medium containing testosterone (1 µM), Finasteride (10 µM), Saw palmetto (100 µg/mL) or SC (25, 50 µg/mL) for 72 h. (A) Western blot analysis of AR, PSA , and 5α-reductase type 2.
These are single continuous images and no image fragments (lanes) were spliced together.
The lanes were not rearranged or not spliced next one to another.
All lanes originated from the same original raw image.
In this study, we used the hand made SDS gel (not the already made commercial product). This reason and the difference of the current flow may have a effect on space between the lanes.
We confirm that these blot data are original raw images.

## Slide 2
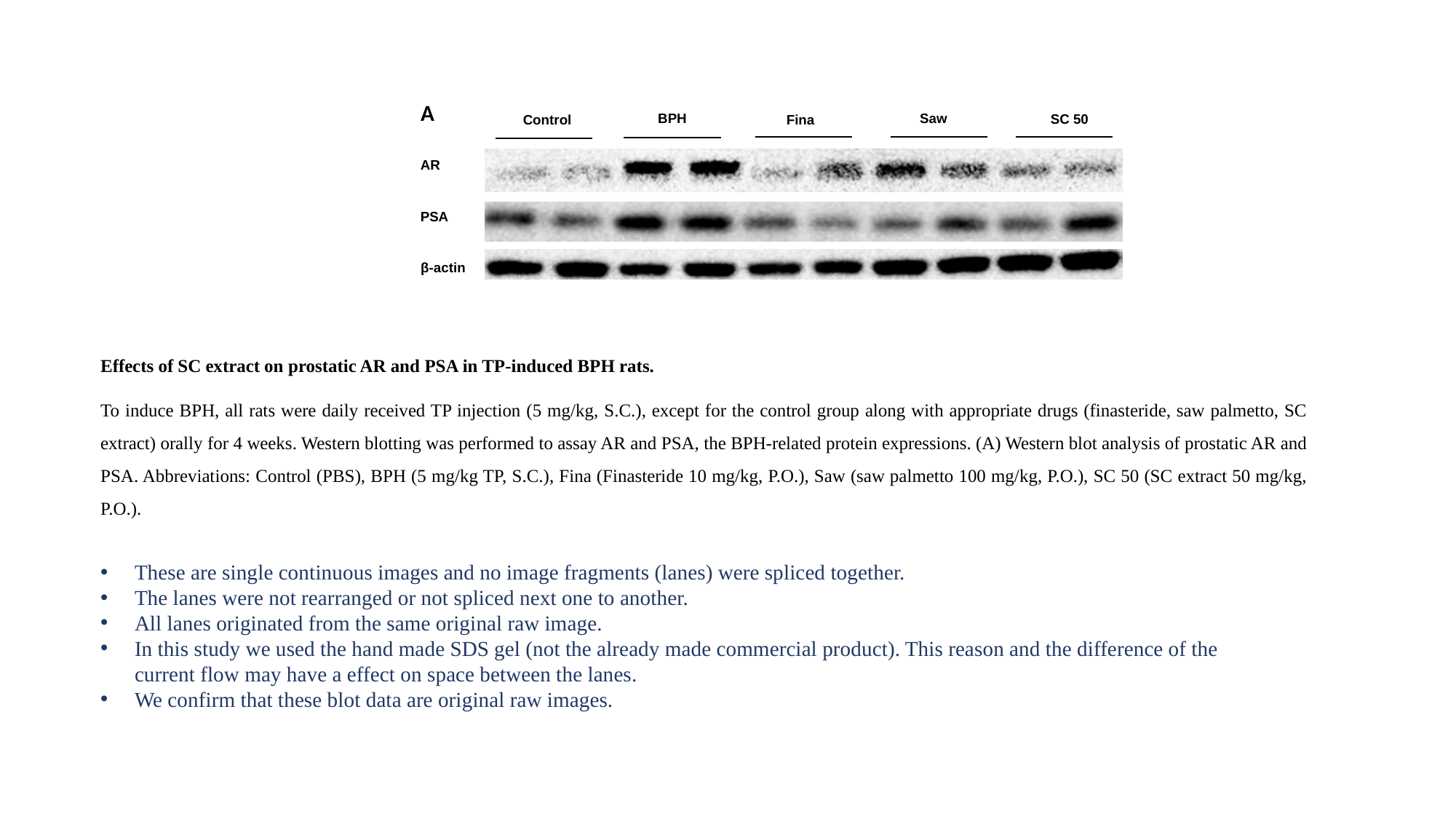

A
Saw
BPH
SC 50
Control
Fina
AR
PSA
β-actin
Effects of SC extract on prostatic AR and PSA in TP-induced BPH rats.
To induce BPH, all rats were daily received TP injection (5 mg/kg, S.C.), except for the control group along with appropriate drugs (finasteride, saw palmetto, SC extract) orally for 4 weeks. Western blotting was performed to assay AR and PSA, the BPH-related protein expressions. (A) Western blot analysis of prostatic AR and PSA. Abbreviations: Control (PBS), BPH (5 mg/kg TP, S.C.), Fina (Finasteride 10 mg/kg, P.O.), Saw (saw palmetto 100 mg/kg, P.O.), SC 50 (SC extract 50 mg/kg, P.O.).
These are single continuous images and no image fragments (lanes) were spliced together.
The lanes were not rearranged or not spliced next one to another.
All lanes originated from the same original raw image.
In this study we used the hand made SDS gel (not the already made commercial product). This reason and the difference of the current flow may have a effect on space between the lanes.
We confirm that these blot data are original raw images.

## Slide 3
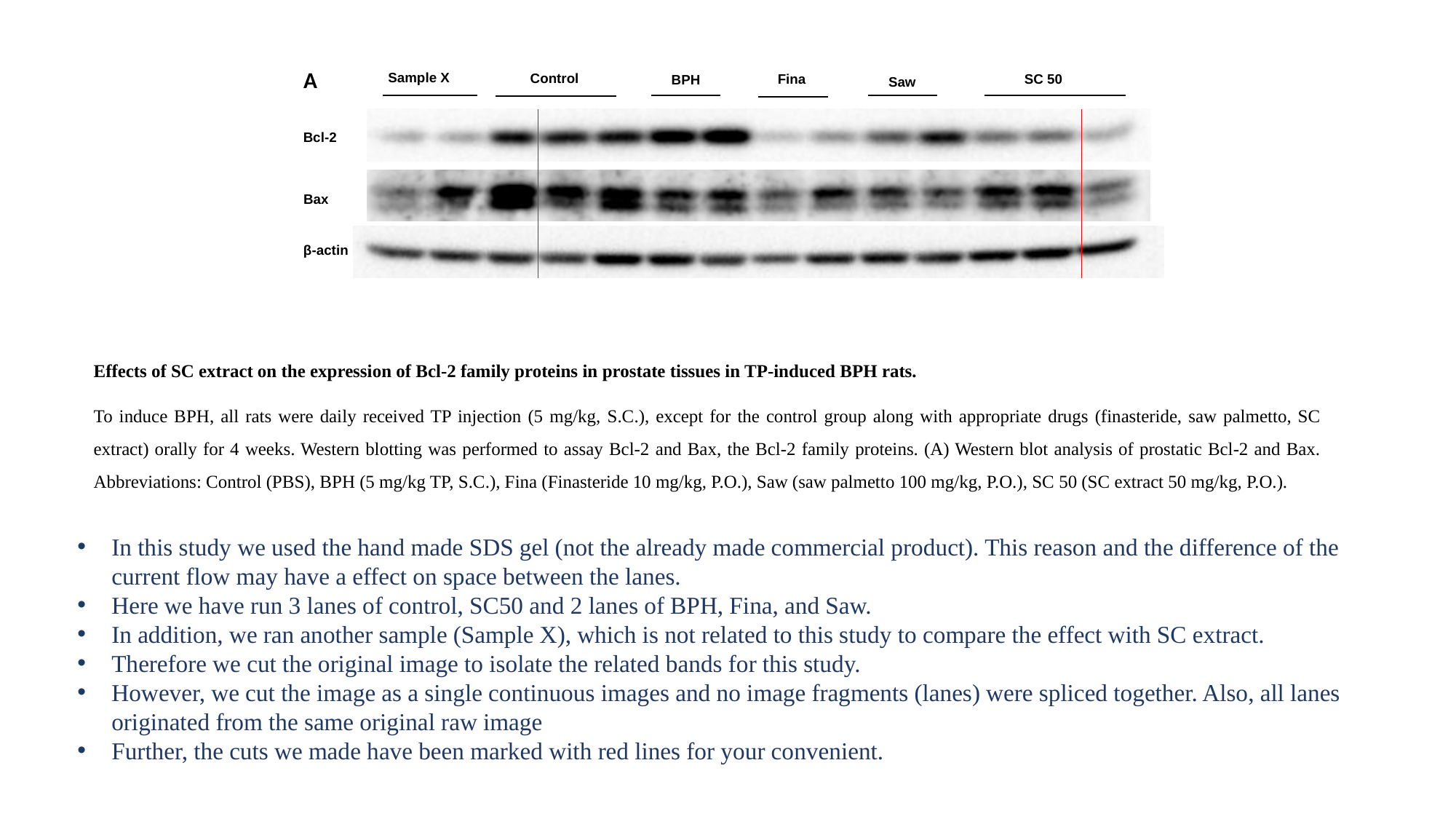

A
Sample X
Control
Fina
SC 50
BPH
Saw
Bcl-2
Bax
β-actin
Effects of SC extract on the expression of Bcl-2 family proteins in prostate tissues in TP-induced BPH rats.
To induce BPH, all rats were daily received TP injection (5 mg/kg, S.C.), except for the control group along with appropriate drugs (finasteride, saw palmetto, SC extract) orally for 4 weeks. Western blotting was performed to assay Bcl-2 and Bax, the Bcl-2 family proteins. (A) Western blot analysis of prostatic Bcl-2 and Bax. Abbreviations: Control (PBS), BPH (5 mg/kg TP, S.C.), Fina (Finasteride 10 mg/kg, P.O.), Saw (saw palmetto 100 mg/kg, P.O.), SC 50 (SC extract 50 mg/kg, P.O.).
In this study we used the hand made SDS gel (not the already made commercial product). This reason and the difference of the current flow may have a effect on space between the lanes.
Here we have run 3 lanes of control, SC50 and 2 lanes of BPH, Fina, and Saw.
In addition, we ran another sample (Sample X), which is not related to this study to compare the effect with SC extract.
Therefore we cut the original image to isolate the related bands for this study.
However, we cut the image as a single continuous images and no image fragments (lanes) were spliced together. Also, all lanes originated from the same original raw image
Further, the cuts we made have been marked with red lines for your convenient.
